# Use of Ecological Momentary Assessment Through a Passive Smartphone-Based App (eB2) by Patients With Schizophrenia: Acceptability Study

**DOI:** 10.2196/26548

**Published:** 2021-07-26

**Authors:** Javier-David Lopez-Morinigo, María Luisa Barrigón, Alejandro Porras-Segovia, Verónica González Ruiz-Ruano, Adela Sánchez Escribano Martínez, Paula Jhoana Escobedo-Aedo, Sergio Sánchez Alonso, Laura Mata Iturralde, Laura Muñoz Lorenzo, Antonio Artés-Rodríguez, Anthony S David, Enrique Baca-García

**Affiliations:** 1 Departamento de Psiquiatria, IIS-Fundación Jiménez Díaz Madrid Spain; 2 Departamento de Psiquiatria, Universidad Autónoma de Madrid Madrid Spain; 3 Centro de Investigación Biomédica en Red de Salud Mental (CIBERSAM) Madrid Spain; 4 Department of Child and Adolescent Psychiatry, Institute of Psychiatry and Mental Health, Hospital General Universitario Gregorio Marañón, IiSGM, CIBERSAM, School of Medicine, Universidad Complutense Madrid Spain; 5 Departamento de Psiquiatria, Hospital Universitario Rey Juan Carlos Móstoles, Madrid Spain; 6 Departamento de Teoría de Señal y de la Comunicación, Universidad Carlos III Madrid Spain; 7 Instituto de Investigación Sanitaria Gregorio Marañón (IiSGM) Madrid Spain; 8 Evidence-Based Behavior Leganés, Madrid Spain; 9 Institute of Mental Health, University College London London United Kingdom; 10 Universidad Católica del Maule Talca Chile; 11 Departamento de Psiquiatría, Hospital Universitario Central de Villalba Madrid Spain; 12 Departamento de Psiquiatría, Hospital Universitario Infanta Elena Valdemoro, Madrid Spain; 13 Université de Nîmes Nimes France

**Keywords:** ecological momentary assessment, acceptability, schizophrenia spectrum disorders, eB2, digital tools, mental health, schizophrenia, real-time data, patients, digital health, internet, mobile apps

## Abstract

**Background:**

Ecological momentary assessment (EMA) tools appear to be useful interventions for collecting real-time data on patients’ behavior and functioning. However, concerns have been voiced regarding the acceptability of EMA among patients with schizophrenia and the factors influencing EMA acceptability.

**Objective:**

The aim of this study was to investigate the acceptability of a passive smartphone-based EMA app, evidence-based behavior (eB2), among patients with schizophrenia spectrum disorders and the putative variables underlying their acceptance.

**Methods:**

The participants in this study were from an ongoing randomized controlled trial (RCT) of metacognitive training, consisting of outpatients with schizophrenia spectrum disorders (F20-29 of 10th revision of the International Statistical Classification of Diseases and Related Health Problems), aged 18-64 years, none of whom received any financial compensation. Those who consented to installation of the eB2 app (users) were compared with those who did not (nonusers) in sociodemographic, clinical, premorbid adjustment, neurocognitive, psychopathological, insight, and metacognitive variables. A multivariable binary logistic regression tested the influence of the above (independent) variables on “being user versus nonuser” (acceptability), which was the main outcome measure.

**Results:**

Out of the 77 RCT participants, 24 (31%) consented to installing eB2, which remained installed till the end of the study (median follow-up 14.50 weeks) in 14 participants (70%). Users were younger and had a higher education level, better premorbid adjustment, better executive function (according to the Trail Making Test), and higher cognitive insight levels (measured with the Beck Cognitive Insight Scale) than nonusers (univariate analyses) although only age (OR 0.93, 95% CI 0.86-0.99; *P*=.048) and early adolescence premorbid adjustment (OR 0.75, 95% CI 0.61-0.93; *P*=.01) survived the multivariable regression model, thus predicting eB2 acceptability.

**Conclusions:**

Acceptability of a passive smartphone-based EMA app among participants with schizophrenia spectrum disorders in this RCT where no participant received financial compensation was, as expected, relatively low, and linked with being young and good premorbid adjustment. Further research should examine how to increase EMA acceptability in patients with schizophrenia spectrum disorders, in particular, older participants and those with poor premorbid adjustment.

**Trial Registration:**

ClinicalTrials.gov NCT04104347; https://clinicaltrials.gov/ct2/show/NCT04104347

## Introduction

Up to 4.1 billion people were reported using the internet in 2019 and 83% of the population worldwide reported having a mobile broadband subscription with internet access, although there were considerable differences between low-income and high-income countries [1]. e-Mental health has become an emerging field for a wide range of mental disorders [2], with a significantly increased number of e-mental health papers published since 1993 [3]. In particular, ecological momentary assessment (EMA) via smartphones (actively or passively) appears to be a clinically useful resource, which enables clinicians and researchers to build a digital phenotype. Digital phenotyping, defined as “the moment-by-moment quantification of the individual-level human phenotype in situ by using data from personal digital devices,” [4] has been applied to a variety of mental disorders [5,6]. In particular, passive EMA, which does not require any active role by the participant, provides 2 significant advantages over traditional data collection methods through validated scales or questionnaires. First, real-time data recording avoids recall bias to which patients are subjected at the time of being interviewed. Second, follow-up face-to-face assessments are not needed, which reduce the risk of attrition and related issues [7].

Schizophrenia can be considered as the most severe mental illness, given its poor clinical and social outcomes [8] and excess mortality [9]. Although EMA has been demonstrated to be a valid and feasible resource in a wide range of mental disorders, including schizophrenia spectrum disorders (SSD) [2], concerns have been raised regarding its safety and acceptability among individuals with SSD. In particular, EMA may exacerbate paranoid thoughts [10], although this remains unclear, and to properly research this, may raise ethical issues [4]. Indeed, concerns about acceptability of EMA methods among patients with SSD have been raised, particularly taking into account the overall poor compliance in patients with SSD [11], which may, however, be improved by EMA-based interventions. In keeping with this, data from a 2016 meta-analysis [12] revealed that up to 66.4% of people with psychosis own a mobile phone, which is likely to have increased by now. Over a decade ago, high levels of acceptance (96%) and compliance (87%) with computerized laboratory methods in patients with schizophrenia and schizoaffective disorders were found, with financial compensation (from US $35 to US $100) provided to participants [13]. Consistent with this, acceptability levels of EMA methods among patients with SSD were relatively good-to-excellent in terms of retention, that is, 92%, according to a 2015 meta-analysis of 5 studies on smartphone-based EMA tools and follow-up periods ranging from 6 to 130 days [14]. However, participants in some of the selected studies received a financial incentive, which may have incorporated a selection bias. In addition, cognitive impairment [15], cannabis use [16], negative symptom severity [17], and clinical appointment attendance [18] were linked with poorer EMA acceptability and compliance in patients with SSD, and illness severity and relapse may lead to exclusion from studies and attrition [19,20]. However, passive smartphone-based EMA apps*,* which require no cooperation from the subject [21], may increase acceptability. Thus, passive EMA metrics, in addition to either active EMA or self-report scales, were reported to be a feasible tool in patients with SSD [20], which can be useful in monitoring clinical improvement during the acute inpatient episode [22] and in assessing a range of illness aspects such as sleep disturbances [23], autonomic deregulation [24], positive and negative emotions [25], symptom severity [21], social stress [26], and functioning [27]. In addition, EMA may aid in predicting clinical outcomes such as transition from “at-high-risk for psychosis” to having a psychotic disorder [28] and relapsing [29]. EMA-based tools such as reminders via text messages may also have a role in treatment [30]. Indeed, a recent meta-analysis, which included 9 studies, showed that EMA improved symptom severity and compliance [19]. Thus, further replication studies testing passive EMA devices in isolation are warranted.

Two previous investigations by our group examined EMA use, acceptability, and compliance, none of which focused on patients with SSD. The first study examined the characteristics of mental health service users who actively used active web-based EMA methods such as MeMind [31] over a 1-year follow-up. Interestingly, out of 13,811 subjects who were registered for MeMind, over 20% of them (2838/13,811) used the active interface on at least one occasion [2]. Later on, a case-control study was designed to test the acceptability of the aforementioned MeMind, which is active (ie, it requires user’s collaboration), and a passive smartphone-based app called the evidence-based behavior (eB2) among patients with mental health disorders with/without a history of suicidal thoughts and behavior and in healthy students (controls) [32]. Regarding the eB2 app, acceptability levels among patients receiving mental health care ranged from 71.7% to 73.5%, although at 2-month follow-up, retention rates dropped to approximately 65% [32].

By building on this work, we aimed to investigate the acceptability of a passive EMA smartphone-based app, eB2, among patients with SSD and what variables predicted this acceptability. Of note, we did not aim to investigate the proportion of patients with SSD who continued using eB2 or the factors influencing the usage of this EMA app. Rather, our research question focused on the extent to which patients with SSD gave consent to installation of a smartphone-based app, which was immediately downloaded by a researcher (VGRR) in front of the patient at that point (ie, acceptability) and the factors underlying this (ie, putative predictors of eB2 acceptability). Specifically, 3 hypotheses were tested. Based on the aforementioned study [32], we postulated that (1) the proportion of randomized controlled trial (RCT) participants consenting to eB2 will be lower than 50% (ie, low recruitment levels) and (2) only a small proportion of eB2 users will continue using the app till the end of the study period (ie, low retention). In addition, we hypothesized that eB2 users (compared with nonusers) will have better neurocognitive and metacognitive performance, less severe psychopathological symptoms, and greater insight levels, thus emerging as predictors of EMA acceptability among patients with SSD and, somewhat, replicating previous findings from our group regarding the acceptability of MeMind [2].

## Methods

### Sample Population

The participants in this study were from an ongoing RCT of metacognitive training, which is being carried out at the Hospital Universitario Fundación Jiménez Díaz (Madrid, Spain) [33]. Briefly, these outpatients (age 18-64 years) with an SSD diagnosis (F20-29 of 10th revision of the International Statistical Classification of Diseases and Related Health Problems), according to the Mini International Neuropsychiatric Interview, 5th edition [34], from June 10, 2019 to March 11, 2020, were considered to be eligible. Recruitment had to be stopped on March 11, 2020 due to the COVID-19 outbreak in Spain. Exclusion criteria were (1) an intelligence quotient ≤70, which was assessed with the short form of the Wechsler Adults Intelligence Scale-IV [35]; (2) a history of head injury or a neurological condition; (3) having received a metacognitive intervention within the previous year; (4) low level of communication in Spanish; and (5) clinician judgment of the participant being unable to complete all aspects of the RCT (eg, clinician-perceived cognitive difficulty of the patient to complete all assessment procedures or weekly therapy group sessions). Of relevance, eligible candidates were reassured that refusing to participate in or dropping out of the study at any time would have no implications on care provision. While customary in research projects, financial compensation of participants may prevent us from fully understanding the acceptability of EMA apps in real-world conditions. In particular, it should be noted that the vast majority of patients with mental health problems receive publicly funded care in Spain, which is free at the point of delivery. Although participants may have been financially compensated for their time, we considered that this may have affected the external validity of our results in terms of EMA acceptability, particularly in our setting. This RCT obtained ethical approval from the local research ethics committee and is registered at clinicaltrials.gov (NCT04104347). Participants gave written informed consent to the research project principal investigator (JDLM) who led the first face-to-face interview with eligible candidates. Those who agreed to participate in the trial were asked if they owned a smartphone. If this was the case, consent to eB2 installation (which is explained below) was attempted to be obtained. Another researcher (VGRR) provided participants in the trial with all the relevant information on eB2, particularly regarding their passive role. More specifically, participants were explained that after installation of the app by one researcher (VGRR) at the clinic in front of them, they did not have to upload data. Equally, they were informed about how to uninstall the app at any time and were provided with the team telephone number. Those who consented to eB2 (users) and those who did not (nonusers) were compared on sociodemographic, clinical, neurocognitive, psychopathological, insight, and metacognitive variables as putative predictors of eB2 acceptability.

### eB2 App

The eB2 [36] is a “passive” smartphone-based platform [37] available on Android and iPhone operating systems, designed for recording functioning-related data such as mobility (location, distance, speed), physical activity (number of steps), sleep data, and social activity (phone use, active apps, social network data) without the subject’s collaboration, that is, running in the background of users’ phones (it is passive) other than the initial configuration, which was assisted by one researcher (VGRR). All the above information is gathered from inertial sensors, physical activity, phone calls, message logs, app usage, nearby Bluetooth and Wi-Fi connections, and location. In addition, more detailed activity information and nearby location data can be accessed through Google Play services. Additional resources include a noncontinuous recording schedule and automatic sleep/wake function so that the battery can be safely saved. Further, if eB2 is stopped due to user’s actions or failures/reboots, the operating system can relaunch itself. Data are anonymized and sent to a secure server, thereby allowing continuous feedbacks from digital phenotyping [5].

### Measures

In terms of sociodemographic variables, we collected data on age, gender, and education level. Regarding clinical variables, diagnosis (schizophrenia-F20 versus all other SSD), previous suicidal behavior (present vs absent), duration of illness (≤5 years vs >5 years), and previous admissions were included. Premorbid adjustment was measured with the premorbid adjustment scale (PAS) [38], which provides 3 scores on childhood, early adolescence, and late adolescence premorbid adjustment (higher scores indicating poorer premorbid adjustment).

Two neurocognitive measures were used, namely, intelligence quotient, which was estimated with the vocabulary subtest of the Wechsler Adults Intelligence Scale-IV [35], and executive function, which was evaluated using the Trail Making Test (TMT) [39]. TMT involves connecting numbers (Task A) or alternating numbers and letters (Task B); therefore, time to complete each task (in seconds) was taken. Subtracting the time to complete task A from time to complete task B provides an overall measure of the executive function (set shifting), having controlled for processing speed (TMT B-A). Psychopathological symptoms were rated using the Spanish version [40] of the Positive and Negative Syndrome Scale (PANSS) for schizophrenia [41].

Clinical insight, which was the primary outcome of the RCT [33], was assessed with the Spanish version [42] of the Schedule for Assessment of Insight [43]; it provides scores on global insight and 3 insight dimensions: illness recognition, symptom relabeling, and treatment compliance. The higher the score, greater the insight. Three metacognitive dimensions were considered, namely, jumping to conclusions (JTC), cognitive insight, and theory of mind (ToM). JTC was measured with the Beads Task [44]. On the basis of probability (in task 1, the probability is 85:15, while in task 2, the probability is 60:40), the individual must decide the jar to which the extracted bead belongs. JTC is considered if a decision is made after extracting 1 or 2 beads. Cognitive insight was measured with the Spanish version [45] of the Beck Cognitive Insight Scale (BCIS) [46], which is a 15-item self-rated scale and yields 2 factors, namely, self-reflectiveness (9 items) and self-certainty (6 items). An overall measure of cognitive insight, that is, composite index, can thus be calculated by subtracting self-certainty from self-reflectiveness. ToM was evaluated by means of the Hinting Task [47] Spanish version [48] by using 2 stories: scores ranged from 0 to 4, where higher scores indicated better ToM performance; and the Emotions Recognition Test Faces activity [49], which is composed of 20 different photographs showing people’s emotions and 2 answers at the bottom of each picture, one of each is right and the other is wrong. Scores ranged from 0 to 20; the higher the scores, the better was the ToM performance.

### Statistical Analysis

First, we calculated acceptability in terms of (1) recruitment, that is, the proportion of RCT participants who consented to eB2 installation (users), and (2) retention, that is, the proportion of users whose eB2 remained installed till the end of the study period. With regard to the predictors of eB2 acceptability, we first inspected the variable distributions by using histograms and the Kolmogorov–Smirnov test. Second, exploratory bivariate analyses investigated differences between users and nonusers in sociodemographic, premorbid adjustment, clinical, neurocognitive, psychopathological, insight, and metacognitive variables. Parametric and nonparametric tests were used as appropriate. Third, a multivariable binary logistic regression model tested the real influence of the above (independent) variables on “being user” (vs nonuser), that is, acceptability, which was the main outcome measure. A 95% significance level was set for all the above analyses, which were performed using SPSS version 25.0 (IBM Corp).

## Results

### Sociodemographic Characteristics of the Participants

**Figure 1:**
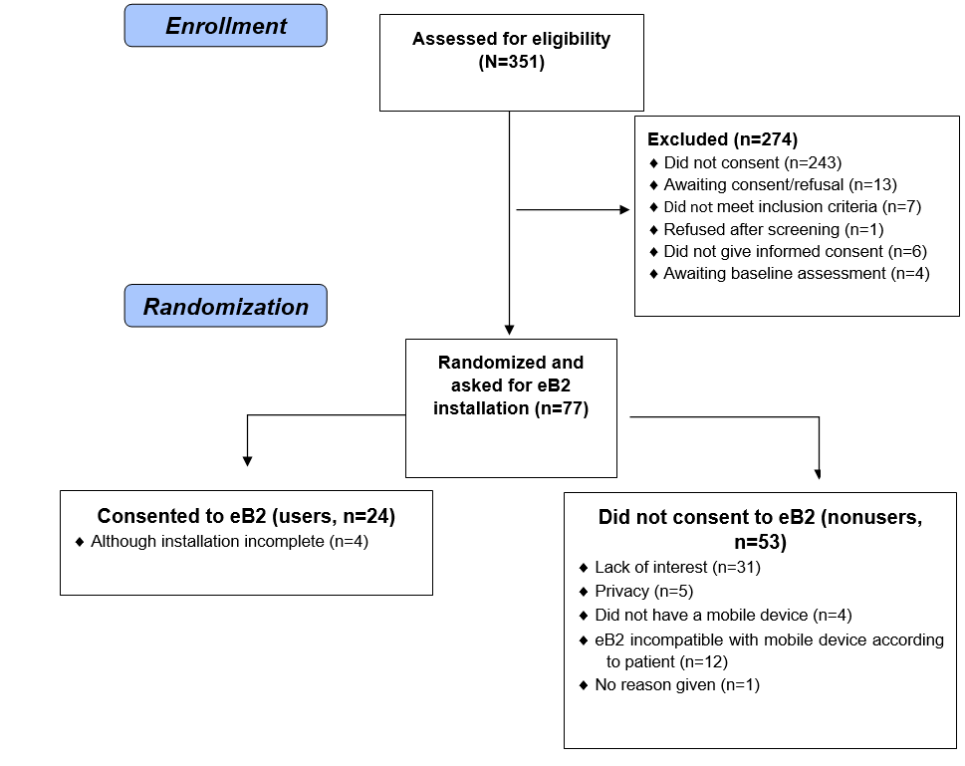
Flowchart in this study. eB2: evidence-based behavior.

From June 10, 2019 to the March 11, 2020, 351 eligible patients were approached and invited to participate in the RCT by the treating consultant psychiatrist (LMI or SSA) or psychologist (LML). Of these, 77 individuals (22%) agreed to take part in the RCT, met the inclusion criteria, and were available at the baseline assessment; therefore, they were asked to install eB2. Twenty-four subjects (31%) agreed and gave consent (users) (Figure 1).

Although in 4 users, eB2 installation could not be completed owing to technical issues, in the analyses below, we compared all the individuals who accepted eB2 (users, 24/77, 31%) with those who did not (nonusers*,* 53/77, 69%). Only 6 subjects uninstalled the app prior to the end of the study, which occurred at a median of 14.50 weeks. Hence, there were 14 users who did not uninstall eB2 (ie, retention was 14/20, 70%) of whom 7 subjects continued to use the app (ie, data being sent to the server) till the end of the study. For the remaining 7 users, there was no evidence of data being sent to the server (eg, they had no wireless access or the battery may have run out without being replaced). Time of eB2 data uploading ranged from 0 to 44 weeks (median 12 weeks).

We asked nonusers (n=53) for the reasons underlying refusal. For descriptive purposes, 4 categories (see below) were created by 2 researchers (JDLM and VGRR) who independently classified participants’ free-text verbal responses according to such categories. Any inconsistency was resolved by consensus by 2 researchers (EBG and MLB). Results were (1) lack of interest (31/53, 58%), (2) privacy issues (5/53, 9%), (3) lack of a mobile device (4/53, 7%), and (4) eB2 incompatible with mobile device (prior to installation) according to the patient (12/53, 23%). One individual gave no reason. However, no nonuser raised issues about the configuration/installation of eB2, which was carried out in front of him/her by one researcher (VGRR), that is, at no point was the smartphone taken away from the owner. Time from eB2 installation to last eB2 data registration by the server (n=20) ranged from 0 to 553 days (median 187.5). One subject experienced a psychotic relapse and asked the research team to uninstall the app, which was honored. However, no causality association between eB2 installation and mental state relapse could be established. The sample population characteristics (N=77) and differences between users (n=24) and nonusers (n=53) are presented in Table 1.

**Table 1:** Sociodemographic characteristics of the participants.

Characteristics		Total sample (N=77)	Users (n=24)	Nonusers (n=53)	*t* (*df*)	*χ^2^* (*df*)	*P* value
**Sociodemographic variables**							
	Age (years), mean (SD)	47.69 (9.76)	42.33 (10.75)	50.11 (8.30)	–3.46 (75)	N/A^a^	.001
	Gender (males), n (%)	41 (53)	14 (58)	27 (51)	N/A	0.4 (1)	.55
	Education level (primary), n (%)	13 (17)	1 (8)	12 (23)	N/A	4.0 (1)	.045
**Premorbid adjustment [38], mean (SD)**							
	Childhood	5.80 (3.79)	4.96 (2.76)	6.19 (4.15)	–1.53 (64.49)	N/A	.13
	Early adolescence	7.64 (4.64)	5.33 (2.76)	8.71 (4.95)	–3.80 (71.21)	N/A	<.001
	Late adolescence	7.69 (4.90)	6.57 (3.07)	8.23 (5.52)	–1.63 (67.33)	N/A	.11
**Clinical variables**							
	Diagnosis, n (%)	48 (62)	16 (67)	32 (60)	N/A	0.3 (1)	.60
	Previous suicidal behavior, n (%)	31 (40)	9 (37)	22 (41)	N/A	0.1 (1)	.74
	Duration of illness (<5 years), n (%)	8 (10)	2 (8)	6 (11)	N/A	0.2 (1)	.69
	Previous admissions, mean (SD)	3.46 (3.99)	2.96 (2.87)	3.68 (4)	–0.72 (74)	N/A	.47
**Psychopathology (PANSS)^b^** **, mean (SD)**							
	Positive	8.44 (3.67)	9.17 (3.94)	8.11 (3.53)	1.17 (75)	N/A	.25
	Negative	14.91 (5.89)	14.00 (5.27)	15.32 (6.16)	0.50 (75)	N/A	.37
	Disorganization	6.05 (2.61)	5.50 (2.38)	6.30 (2.69)	–1.25 (75)	N/A	.21
	Mania	6.25 (1.86)	6.67 (2.20)	6.06 (1.67)	1.34 (75)	N/A	.18
	Depression	6.94 (2.70)	7.29 (2.77)	6.77 (2.67)	0.78 (75)	N/A	.44
**Insight (SAI-E)^c^** **, mean (SD)**							
	Total insight	15.55 (2.29)	15.67 (5.26)	15.49 (5.35)	0.13 (75)	N/A	.89
	Illness recognition	5.36 (2.68)	5.29 (2.77)	5.40 (2.67)	–0.16 (75)	N/A	.87
	Symptoms relabeling	5.87 (2.81)	6.21 (2.23)	5.72 (3.05)	0.79 (59.61)	N/A	.43
	Treatment compliance	4.31 (1.57)	4.17 (1.58)	4.38 (1.58)	–0.54 (75)	N/A	.59
**Neurocognition, mean (SD)**							
	Intelligence quotient	104.61 (11.72)	105.83 (12.48)	104.06 (11.44)	0.61 (75)	N/A	.54
	Trail Making Test A [39]	59.33 (29.50)	45.38 (12.51)	65.90 (30.16)	–4.16 (69)	N/A	<.001
	Trail Making Test B	126.25 (56.83)	102.13 (34.27)	138.57 (62.20)	–3.18 (63)	N/A	.002
	Trail Making Test B–A	68.91 (43.65)	56.75 (34.68)	75.13 (46.70)	–1.70 (63)	N/A	.09
**Metacognition**							
	Jumping to conclusions (85:15), n (%)	42 (56)	13 (54)	29 (54)	N/A	0.1 (1)	.83
	BCIS-SR^d^, mean (SD)	15.43 (5.11)	17.18 (3.86)	14.69 (5.41)	1.95 (72)	N/A	.06
	BCIS-SC^e^, mean (SD)	7.67 (3.42)	7.42 (3.20)	7.80 (3.55)	–0.44 (71)	N/A	.66
	BCIS-CI^f^, mean (SD)	7.74 (6.66)	9.95 (4.789	6.73 (7.17)	1.92 (68)	N/A	.06
	Hinting task, mean (SD)	2.25 (1.33)	2.08 (1.38)	2.32 (1.31)	–0.72 (75)	N/A	.47
	ERTF^g^, mean (SD)	16.86 (2.16)	16.96 (1.90)	16.81 (2.28)	0.27 (75)	N/A	.79

^a^N/A: not applicable.

^b^PANSS: Positive and Negative Syndrome Scale for schizophrenia [41].

^c^SAI-E: Schedule for Assessment of Insight, expanded version [43].

^d^BCIS-SR: Beck Cognitive Insight Scale-Self-Reflectiveness.

^e^BCIS-SC: Beck Cognitive Insight Scale-Self-Certainty.

^f^BCIS-CI: Beck Cognitive Insight Scale-Cognitive Insight.

^g^ERTF: Emotions Recognition Test Faces [49].

At the time of the study inception, users were younger than nonusers (42.33 [SD 10.75] vs 47.69 [SD 9.76] years, respectively; *t_75_*=–3.46; *P*=.001). The proportion of less educated people among users was lower than that among nonusers (1/24, 8% vs 8/53, 23%, respectively; *χ^2^_1_*=4.0; *P*=.045). No between-group differences in gender, diagnosis, history of suicidal behavior, illness duration, or number of previous admissions emerged from the analyses. PAS scores among users were lower (ie, better premorbid adjustment) than those among nonusers, which reached significance in early adolescence (5.33 [SD 2.76] vs 8.71 [SD 4.95], respectively; *t_71.21_*=–3.80; *P*<.001). No significant differences between users and nonusers in psychopathological symptom severity (PANSS factors) or clinical insight scores were found. Although intelligence quotient did not significantly differ between groups, users had better executive function performance, that is, it took them shorter time (in seconds) to complete TMT-A (45.38 [SD 12.51] vs 65.90 [SD 30.16], respectively; *t_63_*=–4.16; *P*<.001) and TMT-B (102.13 [SD 34.27] vs 138.57 [SD 62.20], respectively; *t_63_*=–3.18; *P*=.002) than nonusers. Differences in time to complete task B minus time to complete task A were nonsignificant (*P*=.09). In terms of metacognitive tasks, the BCIS self-reflectiveness (17.18 [SD 3.86] vs 14.69 [SD 5.41], respectively; *t_72_*=1.95; *P*=.06) and the BCIS Composite Index (9.95 [SD 4.78] vs 6.73 [SD 7.17], respectively; *t_68_*=1.92; *P*=.06) among users were higher than those among nonusers (indicating better metacognitive performance). Neither JTC (*P*=.83) nor ToM measures (Hinting Task: *P*=.47; Emotions Recognition Test Faces activity: *P*=.79) distinguished users from nonusers significantly.

### Binary Multivariable Logistic Regression Model on User (as Outcome)

Age, education level, early adolescence premorbid adjustment, TMT-A, TMT-B, and cognitive insight (only composite index was taken to avoid multicollinearity) were significantly associated with being a user, and they were therefore entered into the binary multivariable logistic regression model (Table 2).

**Table 2:** Multivariable binary logistic regression model.^a^

Characteristics	Unstandardized coefficient	SE	Wald	*P* value	Odds ratio (95% CI)
Age	–0.075	0.038	3.910	.048	0.928 (0.861-0.999)
Education level	–0.967	1.289	0.563	.45	0.380 (0.030-4.755)
Early premorbid adjustment scale [38]	–0.285	0.110	6.695	.01	0.752 (0.606-0.933)
Trail Making Test A [39]	–0.030	0.025	1.488	.22	0.970 (0.924-1.018)
Trail Making Test B	–0.005	0.010	0.278	.60	0.995 (0.976-1.014)
Cognitive insight	0.062	0.061	1.043	.31	1.064 (0.944-1.200)

^a^Model *χ^2^*_6_= 25.3, *P*<.001. The model explained 44.7% (Nagelkerke *R*^2^) of the variance and correctly classified 77% (59/77) of the cases. Specifically, 55% (13/24) of users and 88% (47/53) of nonusers were correctly predicted by the model.

Age (OR 0.93, 95% CI 0.86-0.99; *P*=.048) and early adolescence PAS score (OR 0.75, 95% CI 0.61-0.93; *P*=.01) remained significant. The final model (*χ^2^*_6_=25.3; *P*<.001) explained 44.7% (Nagelkerke R^2^) of the variance on being a user (or acceptability, ie, the outcome variable) and correctly classified 77% (59/77) of the subjects, that is, 54% (13/24) of the users and 88% (47/53) of the nonusers (Table 2).

## Discussion

### Main Findings

We used data from an ongoing RCT of metacognitive training with an unselected sample of patients with SSD [33] and we compared those participants who consented to installation (users) of a passive smartphone-based EMA app, eB2, with those who did not (nonusers) in order to investigate eB2 acceptability and the factors that predicted the acceptability. First, as postulated, acceptability was lower than 50% (approximately one-third of the participants), thereby indicating low recruitment. However, contrary to our second hypothesis, retention was higher than expected since most users had not uninstalled eB2 till the end of the follow-up period (at 14 weeks) and in half of the users, there was evidence of continued eB2 use. However, this may have been due to the chronicity of the participants (illness duration was longer than 5 years in almost 90% of them) or negative symptom severity (based on the PANSS ratings). In other words, not having uninstalled the app may well reflect a lack of interest in eB2 rather than the other way round. Our third hypothesis was in part supported by the study results. eB2 users were younger, had a higher education level, better premorbid adjustment and executive function, and higher levels of cognitive insight than nonusers, although only being young and good early adolescence premorbid adjustment survived the multivariable regression model.

### Comparison With Previous Literature

#### Acceptability of eB2 Among Patients With SSD

Previous studies on mobile-based apps in schizophrenia showed good acceptability levels. Back in 2013, a mobile app and text messaging system was tested in 24 subjects: patients with schizophrenia (n=22) and patients with schizoaffective disorder (n=2) (Diagnostic and Statistical Manual of Mental Disorders, 4th Edition criteria), among whom recruitment rate was estimated at approximately 70% [50]. Consistent with this, exploring the mental health app FOCUS use by 33 individuals with schizophrenia was reported to be 61% [51]. However, participants in these studies received a financial incentive, which may raise ethical issues [4], particularly in patients with serious mental illness such as SSD, who tend to have limited incomes [52-54]. Hence, owing to a potential selection bias, caution is needed when interpreting these findings. In keeping with this, acceptability of a smartphone-based app designed for self-reporting psychotic symptoms was significantly lower (50%) when there were no financial incentives [7].

Of note, all the above studies required an active role by participants who had to upload data to the apps themselves. Since patients with SSD tend to have poor compliance [11,55], an alternative to increase EMA acceptability may be using passive smartphone-based apps such as eB2. A passive smartphone EMA tool correlated with in-clinic assessments of sleep quality, including high retention levels (90%) over 6 weeks [23]. Another passive approach to recording functioning data is wearing mHealth devices, which was accepted by the vast majority (80%) of those inpatients with schizophrenia who were found eligible in one study [20]. Consistent with this, 14 out of 15 participants completed an investigation (ie, high retention levels, 93%) on rest/activity recording at sleep time (recruitment was not reported) who received a financial compensation [56]. Regarding patient satisfaction, it is worth noting that 81% of a small sample of patients (n=30) with schizophrenia wearing these devices provided positive or excellent feedback [24].

One may question why we decided to test a passive EMA app in this unselected sample of patients with SSD. As mentioned above, the main aim of this investigation was to test whether metacognitive training can improve insight and clinical outcomes in patients with SSD, that is, addressing noncompliance, which is of major relevance in the psychosis field. In keeping with this, we speculated that a passive EMA app (which required no actions to be taken by the subject) would achieve higher levels of acceptance than an active app. Given the comprehensiveness of the RCT, we considered that asking participants to install an app and upload data (ie, active apps) would make refusal more likely. However, whether passive or active EMA devices may increase or decrease acceptability among patients with psychotic disorders requires further investigation. The extent to which EMA devices may trigger psychotic phenomena in some, but not all, individuals [10] may, in part, explain this. Future studies, free of financial incentives to participants, are therefore warranted to establish what determines this response, including between-individual differences.

#### Predictors of Acceptability of eB2 Among Patients With SSD

In our sample, users were significantly younger than nonusers. At first glance, one may question the extent to which a between-group difference of 8 years, although statistically significant, is clinically meaningful. In this regard, 2 issues should be noted. First, this finding is in full agreement with that reported in our previous study [2], which found a difference in the age by 6 years between a large group (n=2838) of active users of an active EMA tool, such as MeMind, and nonusers (n=10,973). Second, based on the nQuery method, this difference equates to an effect size of 0.79, that is, a large effect size, which would provide further support for this finding. It is true, however, that the full clinical implications of this result remain subject to further debate. Although it is intuitive to think that youth may be linked with higher levels of eHealth literacy, which may contribute to EMA acceptability, smartphone-based EMA has been successfully implemented in older adults with schizophrenia [57]. Our findings also revealed that users have higher education, better premorbid adjustment, and better executive function performance than nonusers, which is consistent with that reported in a previous study showing a positive association of EMA compliance with neurocognition [15].

Although some previous studies linked noncompliance with negative symptom severity and low premorbid intelligence quotient [17], we failed to replicate this. However, our results were consistent with those of other studies that showed no association of EMA compliance with clinical variables [58] or age, medication, and symptom severity [59]. In line with our results, refusal to participate in one study [18] was associated with clinical appointment nonattendance. Of relevance, while no clinical insight dimension such as illness awareness, symptoms, relabeling, and treatment compliance [60] differed between users and nonusers, cognitive insight was found to predict eB2 acceptability in the bivariate analyses. Cognitive insight [46], which is a core metacognitive domain, may thus become a more relevant predictor of EMA acceptability among subjects with SSD than clinical insight despite the relationship between clinical insight and compliance [11,55]. If the aforementioned association of cognitive insight with EMA acceptability/compliance was replicated, interventions targeting metacognition, such as metacognitive training [61], may increase EMA acceptability/compliance, which remains to be investigated.

### Strengths, Limitations, and Implications on Clinical Practice and Future Research

In spite of previous concerns about EMA tool use by patients with SSD, almost one-third of the trial participants agreed to the installation of the eB2 passive mobile app, which opens new directions for clinical practice and research. Participants did not have to take any active role in uploading data or installing the app, which was completed by one researcher (VGRR), which may have increased acceptability levels. EMA-based methods may also pave the way toward remote monitoring of such a vulnerable group of patients. However, further studies should explore more successful strategies aimed at increasing EMA acceptability among patients with SSD. Information leaflets explaining evidence-based benefits from EMA in lay terms within a proper patient-researcher/clinician relationship may contribute to this. Unlike most previous studies [14], participants in this RCT did not receive a financial compensation. Although subject to further debate, by doing so, we may have avoided a potential selection bias. In other words, our findings do reflect the extent to which real-world patients with SSD consented to a mobile-based EMA app, and what appears to underlie this, regardless of the potential financial incentives. Specifically, the vast majority (31/53, 59%) of those who refused to install eB2 reported lack of interest. Although a financial incentive may have reduced the proportion of nonusers, this would have not reflected a real patient involvement in using EMA.

This study findings should be considered in light of some limitations. First, participants came from an RCT and therefore gave consent and completed a comprehensive set of assessments. Lack of cooperativeness was also an exclusion criterion. Thus, referrers (LLI, SSA, LML) only found 351 patients to be eligible over the study recruitment period, which had to be stopped owing to the COVID-19 outbreak in Spain. Regretfully, we did not systematically record the total number of patients that they saw in clinic, which was much higher. Only 77 agreed to take part in the RCT. Hence, those with poorer insight levels were therefore less likely to take part in this study, which may limit the generalizability of the results. However, this ethical requirement and the subsequent limitation in terms of generalizability applies to most studies on insight in psychosis. Nonetheless, this study is part of an RCT of metacognitive training (detailed above), which was not originally designed to test this study hypotheses. Not only this may have affected the representativeness of the study sample, but also much caution should be taken when applying the study results to patients with SSD in other settings. Second, all participants were mental health service users living in Madrid, which is an inner urban area, and results may not apply to people with psychosis receiving mental health input from primary care (ie, only from the general practitioner) or those residing in rural areas. Third, only a small proportion of the RCT participants agreed to eB2 installation; therefore, some between-group (users vs nonusers) comparisons may have lacked sufficient power. Fourth, other nontested variables may affect eB2 acceptability. Finally, future studies may involve families and carers in EMA app installation and compliance. Regretfully, we did not systematically collect data in the first face-to-face interview with participants in the RCT in terms of variables related to the researchers asking for consent (JDLM and VGRR) and whether their relationship with the participants may have affected eB2 acceptability. This said, we suggest registering information on these variables in future EMA acceptability studies. Further, it should be noted that as per the protocol of the RCT, these EMA users had to have consented to participating in the trial. Hence, we cannot rule out that a number of those who refused to enroll the RCT may have accepted eB2, although this seems unlikely since RCT participants tend to be those individuals with higher levels of cooperativeness.

Our work, therefore, adds to the growing field of e-mental health. Within the context of the COVID-19 pandemic, remote telemedicine-based mental health services need to be prioritized, which is in line with previous mental health policies focused on information and communication technology [62]. Not only the COVID-19 pandemic is likely to have a negative impact on mental health outcomes, including increased suicide rates [63] via unemployment rise [64], but also underfunded/underresourced services will have to continue delivering mental health care to vulnerable patients in need [65]. Although eHealth tools may mitigate this [66], long-term outcomes remain unknown, particularly regarding patients with SSD.

In keeping with this, our results highlight that EMA methods may need to be tailored to patients with SSD. Otherwise, there is a high risk that this group of vulnerable patients may be neglected by newly developed approaches to mental health care with the subsequent very negative impact on outcomes, including increased stigma. Specifically, much attention should be paid to patients with SSD when getting older and to those with low education level, poor premorbid adjustment, and deficits in executive function and cognitive insight. Worryingly, not only are these the most vulnerable individuals affected by such a serious mental illness, but also, based on our results, they appear to be the most reluctant ones to use remote resources such as EMA, which are likely to be prioritized by mental health services in the post-COVID-19 pandemic era. The question, therefore, arises: how can we practically tailor EMA methods to subjects with SSD? First, as noted above, we think that passive apps should be more widely recommended than active apps, given the overall poor compliance in patients with SSD. Second, patients need to be properly reassured that these resources do not control their thoughts or monitor them personally since data are anonymized, which requires a proper doctor-patient relationship. In other words, patients with SSD may be less likely to consent to researchers who are not involved in their clinical care. This said, this is definitely an area in which further research is needed.

More specifically, 2 main interlinked unmet challenges need to be addressed by the so-called e-mental health in the years to come. First, the satisfaction of the patients with SSD toward, and adherence to, new technologies and related devices should be improved. Second, financial incentives should not be considered to achieve this, which may stress further underfunded mental health services, particularly in low-income countries.

## Abbreviations

BCIS: Beck Cognitive Insight Scale
eB2: evidence-based behavior
EMA: ecological momentary assessment
JTC: jumping to conclusions
PANSS: Positive And Negative Syndrome Scale for schizophrenia
PAS: premorbid adjustment scale
RCT: randomized controlled trial
SSD: schizophrenia spectrum disorders
TMT: trail making test
ToM: theory of mind
